# Graphene Oxide-Induced Toxicity in Social Insects: Study on Ants Through Integrated Analysis of Physiology, Gut Microbiota, and Transcriptome

**DOI:** 10.3390/insects17010104

**Published:** 2026-01-16

**Authors:** Ting Lei, Ziyuan Wang, Xinyu Wang, Shulan Zhao, Li’an Duo

**Affiliations:** College of Life Sciences, Tianjin Normal University, Tianjin 300387, China; leiting0905@163.com (T.L.);

**Keywords:** social ants, graphene oxide, antioxidant network, mitochondrial dysfunction, gut microbiota

## Abstract

Ants act as keystone species in terrestrial ecosystems, providing important ecosystem services. The large-scale production and application of GO constitute a predominant contributor to its inevitable environmental dispersion. Most GO toxicity studies have focused on plants, animals, and microorganisms, with limited research on ground-dwelling ants. In the study, we used *Camponotus japonicus* as a model to investigate the toxic effects of GO on ants by integrating physiological characteristics, gut microbiota and transcriptome profiling. Results showed that GO exposure induced mitochondrial dysfunction, as evidenced by mitochondrial ROS accumulation and elevated mitochondrial membrane permeability. Physiological assessments revealed that GO exposure induced oxidative stress. Specifically, GO treatment significantly suppressed superoxide dismutase (SOD) and catalase (CAT) activities, while enhancing peroxidase (POD) and carboxylesterase (CarE) activities and increasing the levels of malondialdehyde (MDA) and trehalose. Gut microbiota analyses showed that GO remarkably reduced the relative abundance of beneficial bacterial symbionts (e.g., *Candidatus Blochmannia*) and destabilized the whole community structure. Furthermore, transcriptome profiling revealed 680 differentially expressed genes (DEGs) in the ants after GO exposure, most of which were significantly enriched in pathways associated with oxidative phosphorylation. This study suggests that GO may compromise ant-mediated ecosystem function and provides a reference for understanding the environmental risks of GO. Our findings also offer new insights for protecting the ecosystem services of ants.

## Simple Summary

Ants play crucial ecological roles in terrestrial ecosystems, and their colony-based lifestyle makes them beneficial for global ecosystem services. Graphene oxide (GO) is a carbon-based nanomaterial that has been widely used in advanced material engineering, sustainable energy systems, and biomedical technologies. The widespread application of GO raises concerns about its potential toxicity to non-target insects, yet its effects on ants remain largely unexplored. This study investigated the toxic effects of GO on ants. The results indicate that GO exposure induces oxidative stress, disrupts gut microbial homeostasis, and leads to widespread transcriptional dysregulation. Our findings provide new insights into GO-induced toxicity and emphasize the critical need for ecological risk assessment and management of GO contamination.

## 1. Introduction

Social insects are characterized by overlapping generations, reproductive division of labor, and cooperative brood care [1]. Colony-level organization is maintained through chemosensory communication mediated by the transmission, perception, and decoding of semiochemicals [2]. As social insects, ants occupy high trophic levels, play crucial ecological roles, and exhibit a sedentary nesting habit. These characteristics render ants effective bioindicators for assessing environmental pollution and ecosystem health [3]. Their fixity to specific locations allows for long-term monitoring of environmental changes. Moreover, ants act as keystone species in terrestrial ecosystems by promoting soil aeration, seed dispersal, detrital mineralization, and habitat creation, thereby supporting commensal assemblages of other organisms [4]. Through these activities, ants mediate the top-down regulations of biogeochemical nutrient cycling, plant community structure, and ecosystem stability thresholds. Despite their pivotal ecosystem service, the ecotoxicological impacts of graphene oxide (GO) on ants remain largely unexplored.

Graphene oxide (GO) is a two-dimensional engineered carbon-based nanomaterial [5]. Owing to its exceptional properties, including specific surface area, dispersibility, and mechanical strength, GO has been adopted across multiple fields such as advanced material engineering, sustainable energy systems, and biomedical technologies [6,7,8,9]. Such widespread application may substantially increase the likelihood of GO release throughout its entire life cycle from synthesis and industrial use to end-of-life disposal [10]. Accordingly, organisms may be exposed to GO via multiple environmental pathways, including food chains, respiratory deposition, and transdermal absorption [11]. Given the potential for GO accumulation in the environment, its ecotoxicological effects have been studied across diverse taxa [12].

Specifically, experimental evidence suggests that GO exposure elicits time- and dose-dependent ecotoxicological effects in animal models, disrupting key developmental processes. In zebrafish, toxicological assessments have shown that GO exposure can induce oxidative damage and histopathological alterations, and significantly modulate molecular markers associated with development and antioxidant defense [13,14]. Similarly, in earthworms, GO exposure has been found to elicit oxidative stress, lipid peroxidation, lysosomal membrane destabilization, and substantial DNA damage [15]. In plants, GO exposure has been reported to promote biomass accumulation at lower concentrations but induce phytotoxicity at higher concentrations, as evidenced by inhibited photosynthesis, impaired nutrient uptake, and cellular damage [16,17]. GO has also been shown to exert cytotoxicity toward both prokaryotic and eukaryotic microbiota, potentially impairing beneficial microbial-mediated processes essential for agroecological and biogeochemical matrices [18,19]. Emerging evidence indicates that nanomaterial exposure can disturb social behaviors in ants, which may translate into reduced ecosystem services. For example, nanoplastics have been reported to impair foraging and digging behaviors in *Camponotus japonicus* [20]. Likewise, diatomite, silica (raspberry-shaped), and multi-walled carbon nanotubes have been reported to adversely affect digging, corpse-removal, and foraging behaviors in the red imported fire ant (*Solenopsis invicta*) [21]. Biochar has been reported to exert toxicity and repel red imported fire ants (*S. invicta*) in a dose-dependent manner [22]. Given that these behaviors underpin colony-level functioning and ecosystem services, nanomaterial-driven behavioral perturbations may scale up to ecological consequences. However, most research on GO toxicity has focused predominantly on organism-level endpoints, largely neglecting social ants that rely on collective intelligence networks. Accordingly, the toxicological responses and mechanisms in social ants exposed to GO require elucidation.

*C. japonicus* is widely distributed across diverse habitats in East Asia and exhibits pronounced polymorphism and a well-defined division of labor. Considering the keystone roles of ants in sustaining colony integrity and ecosystem functioning, exposure to GO may disrupt social organization and potentially cascade into broader ecological consequences. This study used *C. japonicus* as a model to conduct a comprehensive toxicological assessment of GO. The investigation provides the first toxicological evaluation of GO in ants by integrating measurements of biochemical responses, gut microbial community composition, and gene expression profiles. We hypothesize that GO induces oxidative stress, reshapes the gut microbiota, and alters key molecular pathways in ants. The findings underscore the urgent need for managing GO’s environmental release and contribute to a broader understanding of how GO impacts social ants and the ecosystem services they support.

## 2. Materials and Methods

### 2.1. Nanomaterials

GO was purchased from Suzhou Tanfeng Graphene Technology Co., Ltd. (Suzhou, China). The GO consisted of single-layer sheets with a thickness of 0.6–1.0 nm and a lateral size of 0.2–10 μm, and contained abundant oxygen-containing functional groups, including carboxyl, hydroxyl, epoxy, and carbonyl groups. GO exhibited excellent water dispersibility. Before preparing GO-contaminated baits, the GO was dispersed in deionized water and sonicated to ensure uniform dispersion.

### 2.2. Experimental Design

All ants (*C. japonicus*) were collected from Dejiang County, Tongren City, Guizhou Province, China. The collected colony, including the queen, males, workers, and larvae, was maintained in rearing boxes. After retrieving the colony to the lab, the workers of comparable body size and activity were selected and housed in rearing boxes (32 cm × 22 cm × 15 cm; Taizhou Huangyan Meibao Pet Supplies Co., Ltd., Taizhou, China). The inner walls of the rearing boxes were coated with polytetrafluoroethylene (PTFE) to prevent ants from escaping. All boxes were maintained indoors at a temperature of 25 ± 2 °C, humidity of 60 ± 5%, and a light cycle of 14 h light and 10 h dark, with direct sunlight avoided. The ants were acclimated for one week and fed a 4% honey jelly prepared from honey, sterile deionized water, and agar powder. After the acclimation period, the ants of comparable body size and activity were randomly assigned to either the control group or GO treatment groups. All the treatments were replicated three times, and each replicate included 200 workers per rearing box. The control group received the same bait as during acclimation, whereas the treatment groups received the bait supplemented with GO at 0.1% or 0.3% (*w*/*v*), respectively [23].

### 2.3. Determination of Mitochondrial Function

#### 2.3.1. Isolation of Mitochondria

Mitochondria were isolated from 0.1 g of pooled whole-body ants (7 individuals) following the procedure outlined by Nareshkumar et al. [24] with slight modifications. Briefly, the ants were homogenized on an ice bath in a buffer (pH 7.4) containing 0.25 M sucrose, 10 mM Tris-HCl, 1 mM EDTA, 1 mM DTT, and 0.1% bovine serum albumin (BSA). The homogenate was centrifuged at 800× *g* for 10 min at 4 °C. The resulting supernatant was centrifuged at 8000× *g* for 10 min to obtain the mitochondrial pellet. Protein concentration was determined using the Bradford assay with BSA as the standard [25,26].

#### 2.3.2. Measurement of Mitochondrial ROS

ROS levels were measured using probe 2′, 7′-dichloro-dihydrofluorescein diacetate (DCFH-DA), following the method described by Zhang et al. [27]. Briefly, the isolated mitochondria were incubated with 1 μM DCFH-DA and then washed three times with phosphate-buffered saline (PBS). Fluorescence intensity was measured at 488/525 nm (excitation/emission) using the Tecan Spark multimode microplate reader (Tecan, Männedorf, Switzerland). The ROS levels were expressed as fluorescence intensity per mg protein.

#### 2.3.3. Detection of Mitochondrial Membrane Permeability

Mitochondrial membrane permeability was assessed by detecting the opening of the mitochondrial permeability transition pore (mPTP), following the previously reported method with slight modifications [27]. Briefly, the mitochondria pellets were suspended in buffer containing 230 mM mannitol, 70 mM sucrose, 3 mM HEPES, and 8.3 mM sodium succinate (pH 7.4), and the mitochondrial protein concentration was adjusted to 0.3 mg/mL. The absorbance at 540 nm was monitored using the Tecan Spark multimode microplate reader (Tecan).

### 2.4. Determination of Biomarkers

#### 2.4.1. Antioxidant Enzyme Activity Assays

Pooled ants (0.1 g, 7 individuals) were homogenized in 2 mL of ice-cold phosphate buffer (50 mM, pH 7.0), and the homogenate was centrifuged at 12,000× *g* for 10 min at 4 °C. The resulting supernatant was collected for the determination of superoxide dismutase (SOD) and peroxidase (POD) activities, as well as for quantification of malondialdehyde (MDA) and trehalose levels. The protein concentration was determined using the Bradford assay with BSA as the standard.

The SOD activity was assessed using nitroblue tetrazolium (NBT) method outlined by Gülmez et al. [28]. Briefly, 3 mL of the reaction mixture contained 50 mM phosphate buffer (pH 7.8), 60 μM NBT, 60 μM riboflavin, 13 mM methionine, 100 μM EDTA, and 100 μL enzyme extract. The mixture was incubated for 30 min under a white fluorescent light lamp. The absorbance was measured at 560 nm using a Shimadzu UV-2600i spectrophotometer (Kyoto, Japan). One unit of SOD activity was defined as the amount of enzyme causing 50% inhibition of NBT photoreduction and was expressed as U/mg protein.

The POD activity was determined according to the protocols outlined by Ranjith et al. [26] with slight modifications. Briefly, 20 μL of the enzyme extract was mixed with a reagent containing 60 μL of 20 mM guaiacol, 100 μL of 50 mM phosphate buffer (pH 7.0), and 20 μL of 0.1 M H_2_O_2_. The increase in absorbance at 470 nm was measured using the Tecan Spark multimode microplate reader (Tecan). One unit of POD activity was defined as an absorbance change of 0.01 per min.

Commercial kits for CAT (A007-1-1) and CarE (A133-1-1) assays were obtained from Nanjing Jiancheng Bioengineering Institute (Nanjing, China). The activity was measured strictly following the kit instructions.

#### 2.4.2. Malondialdehyde (MDA) Concentration Assays

The MDA concentration was assessed using the thiobarbituric acid reactive substances (TBARS) method [29,30]. The absorbance at 532 nm was measured, and MDA concentration was quantified using a standard curve generated with malondialdehyde tetrabutylammonium salt.

#### 2.4.3. Trehalose Content Assays

The trehalose content was determined following the procedures outlined by Zhang et al. [31]. Anthrone reagent was prepared by dissolving 0.1 g anthrone in 100 mL of 98% sulfuric acid and protected from light. Briefly, 10 μL enzyme extract was mixed with 10 μL of 1% sulfuric acid and incubated at 90 °C for 10 min. After cooling in an ice bath, 10 μL of 30% KOH was added, followed by a second incubation at 90 °C for 10 min. After the mixture was cooled again, 200 μL of anthrone reagent was added, and the mixture was incubated at 90 °C for a final 10 min. The absorbance at 620 nm was measured using the Tecan Spark multimode microplate reader (Tecan). Trehalose content was quantified using a trehalose standard curve and expressed as mg/g fresh weight (FW).

### 2.5. Gut Microbiota Analysis

#### 2.5.1. Gut Dissection and Collection

By day 28, intestines were dissected from ants in the control, 0.1% GO, and 0.3% GO groups for gut microbiota analysis. The whole dissection procedure was carried out under aseptic conditions in the lab. Each ant was anesthetized on ice, surface-sterilized with 75% ethanol for 1 min, and rinsed three times in sterile water. The ants were fixed on a wax plate with the abdomen immersed in sterile 0.9% NaCl, and the abdominal cuticle was removed to expose the gut. The dissected gut was washed with sterile 0.9% NaCl to get rid of adhering attachments and was then gently transferred to a sterile 1.5 mL microcentrifuge tube. For each treatment group, three replicates were prepared, with 20 whole guts pooled per replicate.

#### 2.5.2. DNA Extraction and 16S rRNA Sequencing

Total microbial genomic DNA (gDNA) was extracted from each sample using the TGuide S96 DNA Extraction Kit (DP812; Tiangen Biotechnology, Beijing, China). The V3–V4 region of the bacterial 16S rRNA gene was amplified using primer pairs 338F (5′-ACTCCTACGGGAGGCAGCA-3′) and 806R (5′-GGACTACHVGGGTWTCTAAT-3′). The polymerase chain reaction (PCR) mixture contained 5 ng template DNA, 0.3 μL each primer (10 μM), 5 μL KOD FX Neo Buffer, 2 μL dNTPs (2 mM), 0.2 μL KOD FX Neo polymerase, and ddH_2_O added to a final volume of 10 μL. PCR cycling conditions were as follows: 95 °C for 5 min; 25 cycles at 95 °C for 30 s, 50 °C for 30 s, and 72 °C for 40 s; followed by 72 °C for 7 min and a 4 °C hold. The amplified products were retrieved and purified. Sequencing libraries were constructed and sequenced on Illumina HiSeq 2500 (Beijing Biomarker Technologies Co., Ltd., Beijing, China) to acquire raw reads.

#### 2.5.3. Bioinformatic Analysis of Gut Microbiota

The raw reads were filtered with Trimmomatic v0.33, and primer adaptors were removed using cutadapt v1.9.1 [32,33]. The clean reads were merged based on overlap with USEARCH v10 [34]. Chimeric sequences were detected and removed with UCHIME v4.2. The obtained sequences were clustered into operational taxonomic units (OTUs) with 97% identity using USEARCH. Taxonomy was assigned to representative OTUs using the Naïve Bayesian classifier against the SILVA reference database. Alpha diversity indices (Chao1, ACE, Shannon, Simpson) were calculated using Mothur v1.30.2. Beta diversity was visualized using principal coordinates analysis (PCoA) and partial least squares–discriminant analysis (PLS-DA) based on Bray–Curtis dissimilarities. LEfSe (Linear discriminant analysis Effect Size) was performed to identify differentially abundant taxa between the control and 0.3% groups.

### 2.6. Transcriptome Analysis

Biochemical analysis revealed significant effects in the 0.3% group. Consequently, only samples from the control and 0.3% group were chosen for transcriptome analysis. At the end of the 30-day exposure period, total RNA was extracted from pooled whole-body ants (0.1 g, 7 individuals) using TRIzol, with three independent biological replicates per group. RNA quality and integrity were detected by Agilent 5300, and the obtained high-quality RNA samples were employed to construct sequencing libraries. Sequencing was performed on an Illumina NovaSeq X Plus platform (Illumina, San Diego, CA, USA) to generate raw reads. Adaptor sequences and low-quality bases were removed from the raw data using fastp (v0.19.5), and clean reads were de novo assembled with Trinity (v2.8.5). Differentially expressed genes (DEGs) between the 0.3% GO treatment and control groups were identified using DESeq2 with padj < 0.05 and |log2FC| > 1. Gene Ontology and KEGG pathway analyses were conducted to annotate DEGs and identify enriched biological pathways. RNA-Seq and bioinformatic analyses were performed by Major Biotechnology Co., Ltd. (Shanghai, China).

### 2.7. Differentially Expressed Gene (DEG) Analysis

To validate the transcriptomic data, eight DEGs were selected for RT-qPCR. Accordingly, total RNA was extracted from both the 0.3% GO and the control after 30 days of GO exposure. First-strand cDNA was synthesized using the Mighty Script Plus First Strand cDNA Synthesis Master Mix kit (gDNA digester) (B639252, Sangon Biotech, Shanghai, China), according to the manufacturer’s protocol. RT-qPCR was performed using SGExcel FastSYBR Mixture kit (B532955, Sangon Biotech, Shanghai, China) on QuantStudio™ 1 Plus system (Applied Biosystems, Waltham, MA, USA). The 60S ribosomal protein L18 (*rpl18*) was used as the internal reference gene [35]. The Ct values of *rpl18* were not significantly different between the control and 0.3% GO groups (Welch’s *t*-test, *p* = 0.091), supporting its suitability as a stable internal control. Primer sequences of the DEGs and *rpl18* can be found in Table S1. Relative gene expression levels were calculated using the 2^−∆∆Ct^ method [36]. The qPCR program was as follows: 95 °C for 3 min, 40 cycles of 95 °C for 5 s and 60 °C for 20 s. RT-qPCR was performed with three biological replicates, each measured in three technical replicates.

### 2.8. Statistical Analysis

For biochemical indicators, data are presented as mean ± standard deviation (SD). Statistical differences were analyzed with a Two-tailed unpaired Student’s *t*-test between two groups or one-way ANOVA analysis followed by Tukey’s post hoc test using SPSS 26.0. Differences were considered statistically significant at *p* < 0.05. Based on biomarker results, the integrated biomarker response index version 2 (IBRv2) was used to assess the toxic effects of GO exposure following the method [37]. Graphs were performed using GraphPad Prism 9.5 and Origin 2024.

## 3. Results

### 3.1. Mitochondrial Dysfunction

**Figure 1:**
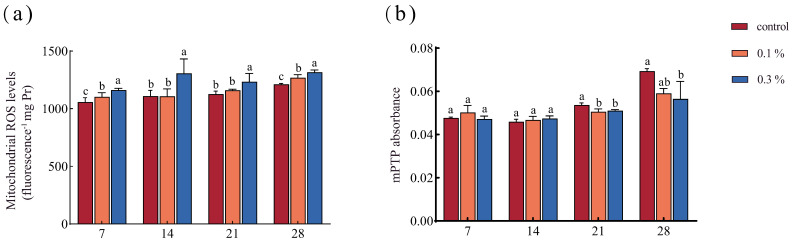
Responses of mitochondria in the ants to GO exposure: (**a**) Mitochondrial ROS; (**b**) Mitochondrial membrane permeability. Each bar represents the mean ± SD. Different letters above the error bars represent significant differences among treatments (*p* < 0.05) (*n* = 3).

GO exposure induced significant time- and dose-dependent mitochondrial toxicity in the ants. By day 7, mitochondrial ROS had already accumulated in GO-exposed ants and were significantly higher than in the control. This trend persisted through days 14, 21, and 28 (Figure 1a). Mitochondrial membrane permeability was evaluated by monitoring mitochondrial permeability transition pore (mPTP) opening at 540 nm. By days 7 and 14, the absorbance of the mitochondria did not differ between control and GO-exposed ants. By day 21, absorbance was significantly lower in both the 0.1% and 0.3% groups than in the control, and by day 28, the 0.3% group showed an 18.46% decrease relative to the control, indicating increased mitochondrial permeability (Figure 1b).

### 3.2. Antioxidant Response

**Figure 2:**
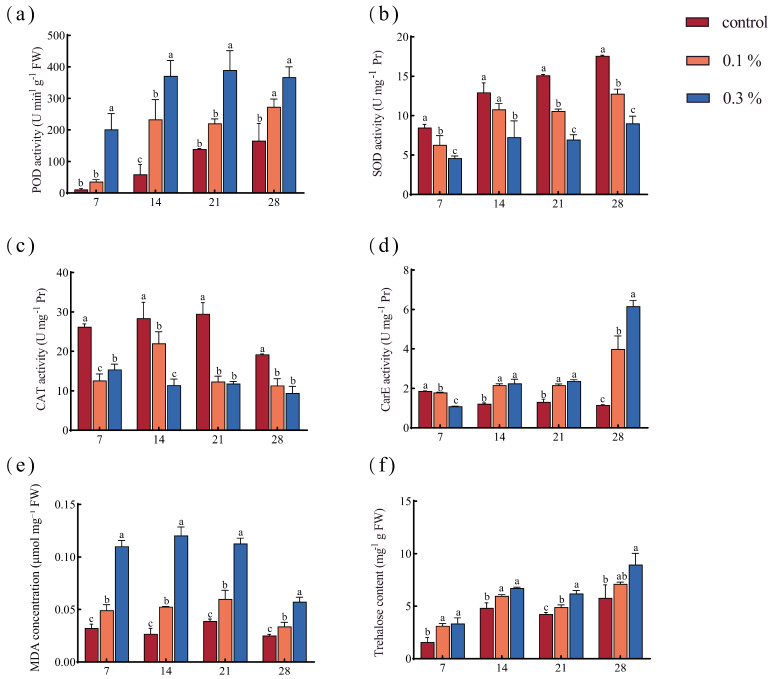
Antioxidant response of ants to GO exposure: (**a**) POD activity; (**b**) SOD activity; (**c**) CAT activity; (**d**) CarE activity; (**e**) MDA concentration; (**f**) trehalose content. Each bar represents the mean ± SD. Different letters above the error bars represent significant differences among treatments (*p* < 0.05) (*n* = 3).

The antioxidant defense system supports essential physiological processes in ants and helps maintain social behavior. During GO exposure, POD, SOD, CAT, and CarE activities, MDA level, and trehalose content were evaluated. POD activity, CarE activity, MDA level, and trehalose content showed significant increases in a dose-dependent manner (Figure 2a,d–f). By day 28, POD activity was 121.6% higher in the 0.3% group than in the control, and CarE activity peaked, showing a 431.4% increase relative to the control. In the 0.3% group, MDA concentration peaked on day 14 and then declined; however, it remained 128.0% higher than the control by day 28. Trehalose content increased with GO concentration and exposure time and remained significantly elevated by 54% in the 0.3% GO group by day 28 compared to the control. In contrast, CAT and SOD activities in the 0.3% GO-treated ants were lower than those of the control on day 7, and this pattern persisted through days 14, 21, and 28. By day 28, CAT and SOD activities in the 0.3% group were reduced by 50.6% and 48.7%, respectively (Figure 2b,c).

### 3.3. IBR Analysis

**Figure 3:**
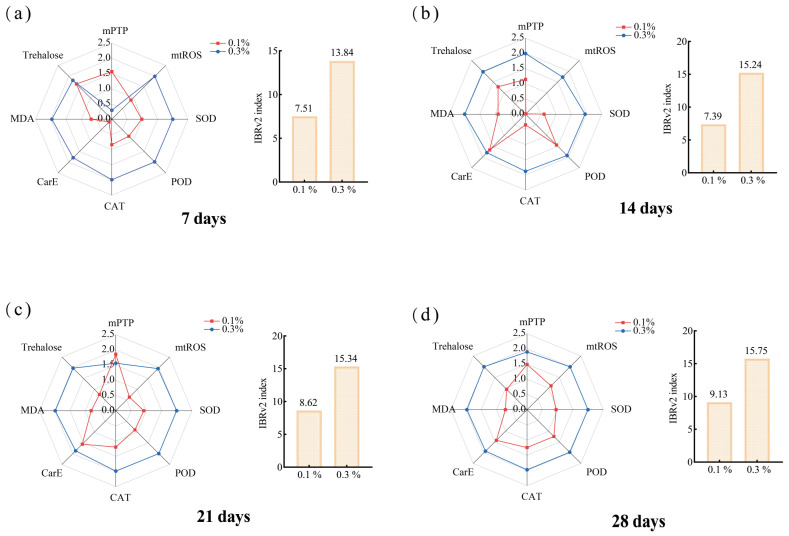
Integrated biomarker response (IBR) analysis of GO-exposed ants at different times: (**a**) 7 days; (**b**) 14 days; (**c**) 21 days; (**d**) 28 days.

Given that the sensitivity of each indicator to pollutants varies, this study combined each biochemical indicator (mitochondrial ROS, mPTP absorbance, POD, SOD, CAT, CarE, MDA, trehalose) for IBR analysis to assess the ecological risk of GO in the ants. Overall, the GO exhibited the time- and dose-dependent toxicity in the ants, and IBRv2 calculated from these indices indicated the highest biological stress in the 0.3% treatment (Figure 3).

### 3.4. Intestinal Microbiota Analysis

The high-throughput sequencing revealed a total of 719,757 valid sequences, with at least 79,414 clean reads per sample. A total of 1325 OTUs were defined based on 97% sequence identity and classified into 28 phyla, 57 classes, 141 orders, 249 families, 560 genera, and 638 species. The rarefaction curves approached saturation for all samples, indicating sufficient sequencing depth for subsequent analysis.

#### 3.4.1. Intestinal Microbiota Diversity

**Figure 4:**
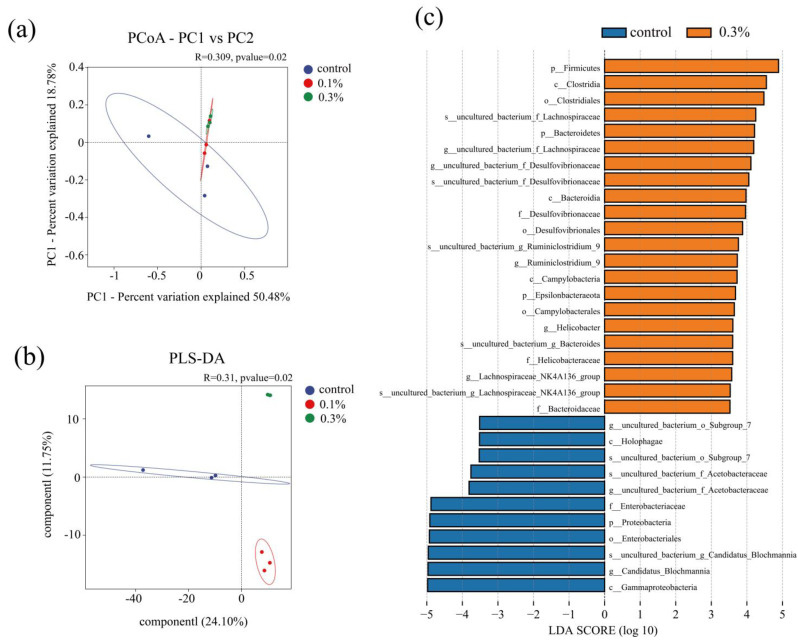
Change in the diversity and LEFSe analysis of gut microbiota after GO exposure: (**a**) Beta diversity indicated by PCoA based on Bray-Curtis distance; (**b**) PLS-DA on OTU level; (**c**) Linear discriminative analysis (LDA) effect size (LEfSe) analysis detected taxonomic differences between the control and 0.3% GO group. Each treatment is presented with different colors and each dot represents an individual sample.

Alpha diversity indices (Chao1, ACE, Shannon, and Simpson) did not differ significantly among groups (Table 1), whereas beta diversity revealed a clear separation of microbial community composition. PCoA based on Bray–Curtis dissimilarity was used to compare the gut community structure, showing that GO-exposed ants differed markedly from the control group (Figure 4a). Consistently, PLS-DA further revealed a clear separation among the control, 0.1% GO and 0.3% GO groups (Figure 4b). The LEfSe algorithm identified several discriminant taxa with an LDA score ≥ 3.5 (Figure 4c). The control group was characterized by the enrichment of the genus *Candidatus Blqchmannia*. In contrast, the 0.3% GO group showed enrichment of multiple taxa, including the genus *Ruminiclostridium_9*, *Helicobacter*, *Bacteroides*, and *Lachnospiracea*_NK4A136_group. Collectively, these results indicate that GO exposure shifted gut bacterial community composition, with distinct biomarker taxa emerging under 0.3% GO.

#### 3.4.2. Intestinal Microbiota Composition

**Figure 5:**
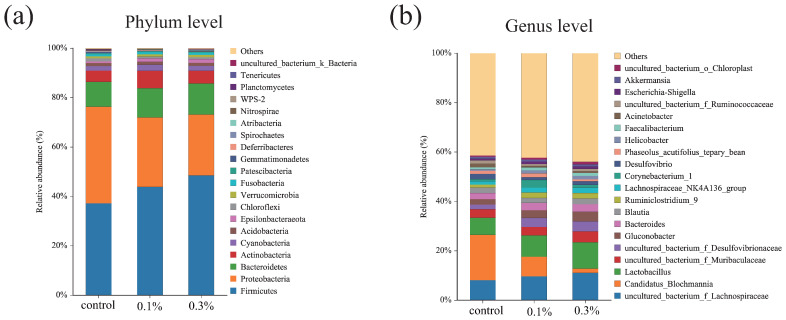
Change in the community composition of gut microbiota after GO exposure: (**a**) Microbiota composition at the phylum level; (**b**) Microbiota composition at the genus level.

To further elucidate the effects of GO on the gut microbiota, a comparative analysis of bacterial community composition was conducted between the control and GO groups. Across all treatments, Firmicutes, Proteobacteria, and Bacteroidetes were the dominant phyla, with Firmicutes being the most abundant (Figure 5a). In the 0.3% GO group, the relative abundances of Firmicutes and Bacteroidetes were higher than in the 0.1% GO group, whereas Proteobacteria were lower than in the control. At the genus level, *Candidatus Blochmannia* showed the highest relative abundance in the control, but the lowest in the 0.3% GO group, whereas *Lactobacillus* exhibited the opposite pattern (Figure 5b).

### 3.5. Transcriptomic Analysis of Ants Exposed to GO

#### 3.5.1. Transcriptome Profiling Data

To further characterize the stress response of ants, transcriptome sequencing was conducted between the control and 0.3% GO-treated groups. Six cDNA libraries were constructed, including three biological replicates for the control group (Ctrl1, Ctrl2 and Ctrl3) and three for the 0.3% GO group (GO1, GO2 and GO3). After adaptor removal and quality filtering, a total of 257,977,888 clean reads were obtained from the six libraries. The number of clean reads generated from the Ctrl 1, Ctrl 2, Ctrl 3, GO 1, GO 2, and GO 3 libraries was 44,500,850, 42,616,148, 44,280,600, 41,051,390, 42,553,902, and 42,974,998, respectively. The proportion of Q30 bases exceeded 94% in all libraries, demonstrating that sequencing quality was sufficient for further analysis.

**Figure 6:**
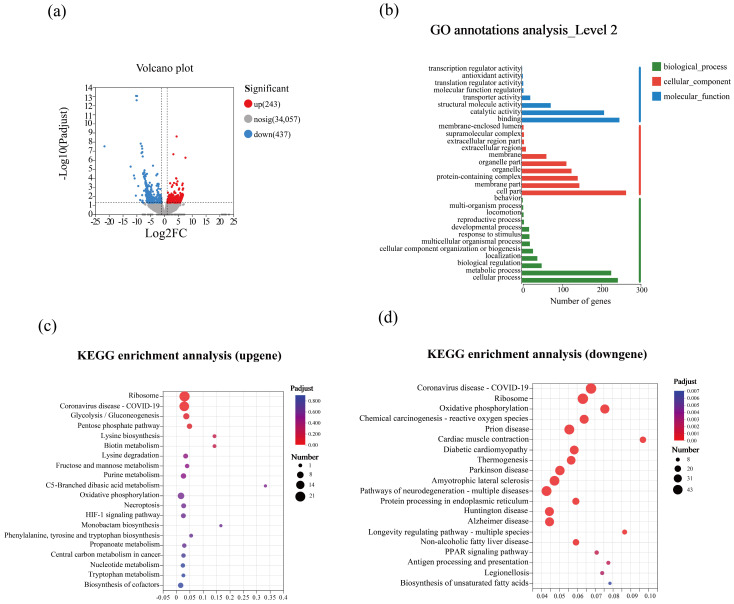
Effects of GO exposure on gene expression and functional analysis of DEGs in the ants after GO exposure. (**a**) Volcano plot showing the DEGs; (**b**) Gene Ontology annotation of the DEGs; (**c**) KEGG enrichment of upregulated genes; (**d**) KEGG enrichment of downregulated genes.

The total number of expressed genes in the control and 0.3% GO was 20,648 and 21,872, respectively. Among these, 5690 and 6914 genes were unique in the control and 0.3% GO groups, respectively, whereas 14,958 genes were shared between the groups. DEGs were visualized through a volcano plot. In total, 680 DEGs were identified between the control and 0.3%, including 243 significantly upregulated and 437 significantly downregulated genes in the 0.3% GO group compared to the control (Figure 6a).

#### 3.5.2. Gene Ontology Annotation and KEGG Pathway Enrichment of DEGs

For functional characterization of the DEGs, Gene Ontology annotation and KEGG pathway enrichment analyses were performed. DEGs were assigned to the three main Gene Ontology categories: biological process (BP), cellular component (CC), and molecular function (MF). At level 2, 40 functional subcategories were identified, indicating a broad transcriptional response to GO exposure (Figure 6b). Within BP, most DEGs were associated with cellular process (245 genes) and metabolic process (228 genes), suggesting that GO primarily perturbs basic cellular activities and metabolism. Within CC, major terms included cell part (266 genes), membrane part (147 genes), protein-containing complex (143 genes), organelle (127 genes), and organelle part (114 genes), implying widespread alterations in cellular components and intracellular structures. Within MF, DEGs were primarily involved in binding (249 genes) and catalytic activity (210 genes), indicating altered expression of binding proteins and enzymes under GO exposure.

KEGG enrichment analysis was then conducted separately for upregulated and downregulated genes, and the top 20 enriched pathways were visualized as a bubble plot. Overall, enriched pathways were mainly related to energy metabolism, carbohydrate metabolism, and environmental adaptation. Among the upregulated DEGs, enriched pathways included glycolysis/gluconeogenesis, pentose phosphate pathway, and oxidative phosphorylation (Figure 6c). Among downregulated DEGs, significant enrichment was observed in oxidative phosphorylation, chemical carcinogenesis–reactive oxygen species, and neurodegeneration–multiple diseases pathways (Figure 6d).

#### 3.5.3. Functional Analysis of DEGs

**Figure 7:**
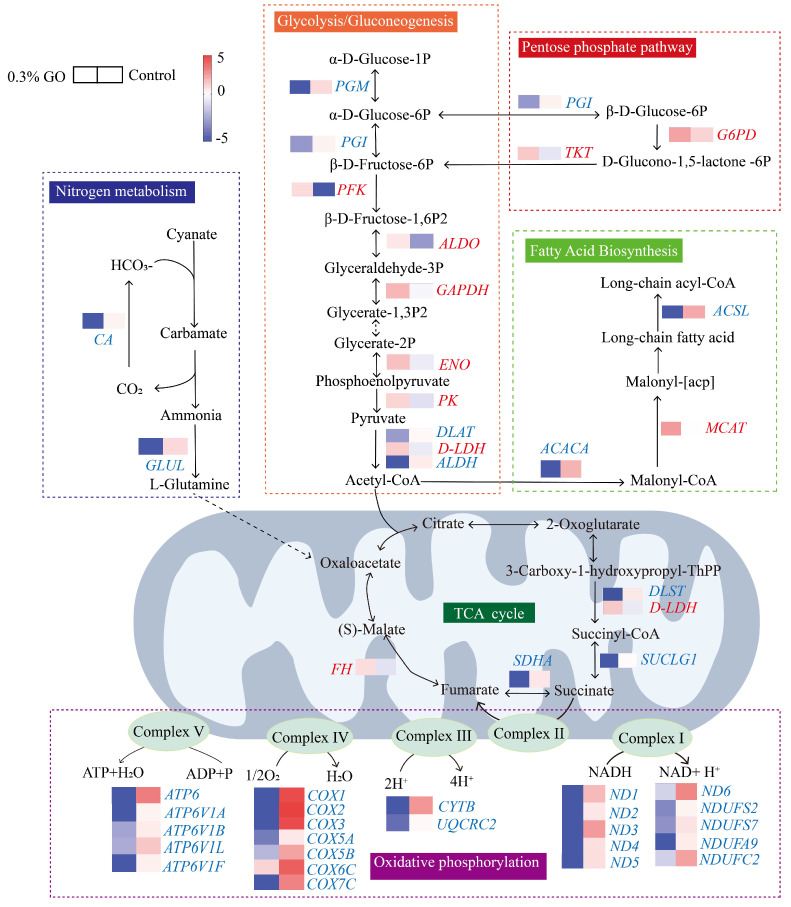
Integrated KEGG pathway map. Different metabolic pathways are enclosed in colored frames. The TCA cycle is depicted within the mitochondrion. Heatmap shows log2 (mean TPM). In the heatmaps, the left bars represent the 0.3% treatment, and the right bars represent the control group. Gene names adjacent to the heatmaps, upregulation of DEGs is labeled in red, and downregulation of DEGs is labeled in blue.

Energy metabolism supports essential biological processes. To elucidate the impact of GO on energy metabolism in the ants, we integrated pathways related to energy metabolism, including glycolysis/gluconeogenesis, pentose phosphate pathway, TCA cycle, oxidative phosphorylation, nitrogen metabolism, and fatty acid biosynthesis (Figure 7). In the ants exposed to 0.3% GO, the expression of key genes involved in oxidative phosphorylation was significantly altered, including genes encoding subunits of complex I (NADH dehydrogenase: *ND1–ND6*, *NDUFS2*, *NDUFS7*, *NDUFA9*, and *NDUFC2*), complex II (succinate dehydrogenase: *SDHA*), complex III (the cytochrome bc_1_ complex: *CYTB* and *UQCRC2*), complex IV (cytochrome c oxidase: *COX1–COX7C*), and complex V (ATP synthase: *ATP6*, *ATP6A*, *ATP6B*, *ATP6F* and *ATP6L*). All DEGs associated with oxidative phosphorylation were downregulated. These changes suggest that GO exposure may impair mitochondrial electron transport chain function and increase energetic stress in the ants. In glycolysis/gluconeogenesis, *PFK*, *ALDO*, *GAPDH*, *ENO*, *PK*, and *D-LDH* were markedly upregulated, whereas *PGM*, *PGI*, *DLAT*, and *ALDH* were downregulated. In the pentose phosphate pathway, *PGI* was downregulated, whereas *G6PD* and *TKT* were upregulated. In the TCA cycle, *FH* and *D-LDH* were upregulated, while *DLST* was downregulated. In nitrogen metabolism, *CA* and *GLUL* were downregulated. In fatty acid biosynthesis, *ACSL* and *ACACA* were downregulated, whereas *MCAT* was upregulated.

### 3.6. Gene Expression Validation by RT-qPCR

**Figure 8:**
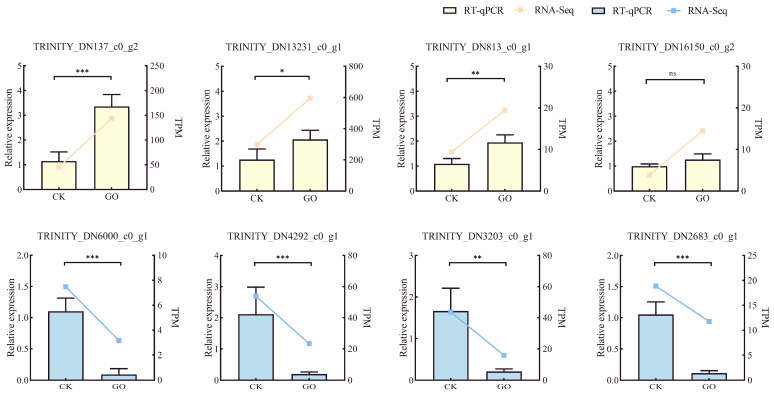
Verification of the relative expression levels of DEGs after GO treatments compared to the control. Error bars in the figure represent the mean ± SD. Notes: * *p*  <  0.05, ** *p*  <  0.01, *** *p*  < 0 .001, ns: *p*  >  0.05.

To validate the RNA-seq results, eight genes associated with energy metabolism, redox regulation, and other key biological processes were selected for RT-qPCR, including the DEGs TRINITY_DN137_c0_g2 (carbohydrate metabolism), TRINITY_DN13231_c0_g1 (oxidoreductase activity), TRINITY_DN16150_c0_g2 (mitochondrial membrane), TRINITY_DN6000_c0_g1 (ion binding), TRINITY_DN2683_c0_g1 (peroxisome), TRINITY_DN3203_c0_g1 (lipid metabolism), TRINITY_DN813_c0_g1 (olfactory transduction), and TRINITY_DN4292_c0_g1 (unannotated). The RT-qPCR results were consistent with the RNA-Seq data, showing similar expression trends for all eight genes (Figure 8).

## 4. Discussion

Considering the inevitable release of GO during its manufacturing and use, its ecotoxicological effects cannot be overlooked. While GO toxicity has been extensively investigated in various organisms, information regarding its impact on social organisms remains limited [38,39,40]. As important ecological engineers in terrestrial ecosystems, ants exert profound effects on soil physicochemical properties and biodiversity through highly organized social behaviors, including nest excavation, food collection and distribution, and symbiotic interactions. These colony-level behaviors ultimately rely on the physiological performance of ants. Accordingly, physiological impairment or energetic stress in ants may scale up to colony-level functional deficits.

### 4.1. GO Exposure Induced Mitochondrial Dysfunction

Mitochondria serve as the primary energy providers within cells. Mitochondria have been recognized as primary targets of environmental toxicants [41]. Mitochondria function not only as a major source of ROS but also as a primary target of ROS-mediated damage. In the present study, GO exposure significantly elevated mitochondrial ROS levels in the ants, indicating that GO elicits oxidative stress. Feng et al. [42] found that GO led to the accumulation of ROS and oxidative stress in SH-SY5Y cells, which was similar to our results. Previous studies have established that the normal opening of mitochondrial permeability transition pore is necessary for maintaining the membrane potential and energy metabolism [43]. When the level of ROS rises to a certain height, ROS in mitochondria leads to increased mitochondrial membrane permeability and the subsequent disruption of energy metabolism. Collectively, our findings indicate that GO exposure induced mitochondrial dysfunction driven by ROS production and the loss of membrane permeability homeostasis. As mitochondria are the main energy-producing organelle, such dysfunction is likely to impose energetic stress on ants and may ultimately compromise their capacity to sustain social behavior.

### 4.2. GO Exposure Induced Oxidative Stress

Antioxidant enzyme systems (such as SOD, CAT, and POD) play a crucial role in maintaining cellular redox homeostasis, thereby protecting lipids, proteins, and DNA from oxidative damage [44,45,46]. SOD catalyzes the dismutation of superoxide anions into dioxygen and hydrogen peroxide, which is subsequently decomposed into water and oxygen by CAT and POD [47,48]. In this study, SOD and CAT activities were inhibited by GO exposure, whereas POD activity was elevated. The marked elevation in POD activity may represent a compensatory response, with POD upregulated to scavenge excess peroxides when SOD and CAT are inhibited. The observed inhibition of SOD and CAT activities may be attributed to the excessive accumulation of ROS, which can induce structural alterations in these enzymes and subsequently impair their catalytic function [49,50]. These alterations in enzyme activity suggest that GO induced oxidative stress and disturbed the antioxidant defense system. Consistent with our findings, previous studies have demonstrated that GO can impair antioxidant enzyme activity in earthworms and crickets [15,51]. Such impairment of the antioxidant enzyme system is expected to compromise ant performance.

Under oxidative stress, excessive ROS attack membrane lipids, leading to lipid peroxidation of MDA, a canonical marker of oxidative damage. The marked elevation in MDA levels following GO exposure indicates enhanced lipid peroxidation and may compromise phospholipid bilayer integrity, thereby impairing membrane structure and physiological performance in the ants. Consistent with Zhao et al., who reported GO-induced MDA perturbation in *Eisenia fetida*, our data exhibit analogous modulatory effects on MDA [15]. Elevated MDA levels reflect oxidative damage to cellular and organellar membranes, which may disrupt membrane transport and signal transduction, and ultimately lead to cellular dysfunction in the ants.

CarE, a key detoxification enzyme, reduces oxidative damage and contributes to defense against oxidative stress [52]. In the present study, CarE activity was significantly elevated by GO exposure, suggesting that GO activated the detoxification pathways to oxidative stress. Trehalose is the predominant storage sugar in insects and serves as a major energy source [53]. Under environmental stress, trehalose acts as a protectant that helps limit oxidative damage [54]. The significant increase in trehalose levels in GO-exposed ants indicates that trehalose contributes to tolerance of GO-induced stress.

### 4.3. GO Exposure Disrupted the Gut Microbial Community

Gut microbiota is crucial for insect survival and ecological performance, contributing to nutrient acquisition, detoxification, immune defense, development, and behavioral adaptation [55]. Stable gut microbial communities are essential for host health, and disruption of this balance can have severe consequences. In insects, Firmicutes, Bacteroidetes, and Proteobacteria represent the dominant bacterial phyla [56]. Similarly, these three phyla were dominant in the gut microbiota of *C. japonicus* in the present study. Under environmental stress, gut microbial dysbiosis has been demonstrated in crickets, cockroaches, and houseflies [51,57,58]. Consistent with these studies, we found that GO exposure altered the gut microbial community composition in the ants: the relative abundances of Firmicutes and Bacteroidetes increased, whereas Proteobacteria decreased in GO-exposed ants. Gut microbial dysbiosis has been associated with body weight loss, delayed development, and mortality. These shifts in this study indicate that GO exposure may impair nutrient acquisition.

In *C. japonicus*, *Candidatus Blochmannia* is an obligatory symbiont that supplies essential amino acids during host development when protein is scarce, as well as tyrosine, which is essential for cuticle synthesis and related processes such as melanization and sclerotization [59]. The reduction of *Candidatus Blochmannia* has been shown to decrease colony growth and weaken immune defenses [60]. In this study, GO exposure decreased the relative abundance of *Candidatus Blochmannia*, which may compromise nutritional status, cuticle quality, and decrease colony growth and weaken immune defenses in the ants. Under environmental stress, suppression of some taxa can create ecological opportunities for others to expand, potentially reshaping gut homeostasis. *Lactobacillus* is widely regarded as a main probiotic species that can regulate the balance of intestinal flora and facilitate resistance to pathogens by the production of antimicrobial compounds [61]. The increased abundance of *Lactobacillus* in GO-exposed ants may reflect a consequence shift following the decline of *Candidatus Blochmannia* and overall community restructuring.

The LEfSe identified *Ruminiclostridium*_9, *Bacteroides*, and *Lachnospiracea*_NK4A136_group as characteristic genera in the 0.3% GO-exposed ants, suggesting that they could serve as biomarkers for GO exposure. *Ruminiclostridium*_9 and *Lachnospiraceae*_NK4A136_group belong to Firmicutes taxa associated with anaerobic fermentation and short-chain fatty acids production, which have been widely associated with host energy supply and gut homeostasis [62,63,64]. *Bacteroides* contribute to nutrient absorption and immune function by fermenting complex carbohydrates and producing beneficial metabolites [65]. The increased abundance of these genera in GO-exposed ants may reflect a microbial shift that helps maintain gut homeostasis.

### 4.4. GO Exposure Altered Transcriptional Regulation of Energy Metabolism

To understand the regulatory mechanisms of ants exposed to GO, transcriptome data were analyzed to identify key affected biological processes. KEGG enrichment analysis showed that DEGs were significantly enriched in oxidative phosphorylation, chemical carcinogenesis–reactive oxygen species, neurodegeneration pathways (Parkinson disease, Huntington disease, Alzheimer disease, and pathways associated with neurodegeneration-multiple diseases), and antigen processing and presentation. Oxidative phosphorylation occurs in the inner mitochondrial membrane and drives ATP production [66]. The overall downregulation of this pathway suggests that GO exposure may compromise mitochondrial energy output, thereby limiting energy available for locomotion, detoxification, and immune responses [67,68]. The chemical carcinogenesis–reactive oxygen pathway is related to oxidative damage [69]. Consistent with these pathways, mitochondrial ROS levels increased markedly, supporting GO-induced oxidative stress. Neurodegeneration pathways, including Parkinson’s, Huntington’s, and Alzheimer’s disease, are associated with behavioral regulation [70,71,72]. The enrichment of these pathways suggests that GO exposure may affect ants’ behavior. Antigen processing and presentation and immune-related pathways were significantly enriched, suggesting immune activation under GO stress [73].

With the exception of the above metabolic processes, we further examined how GO exposure affected energy metabolism and related pathways in the ants. The mitochondrial electron transport chain (ETC) couples electron transfer to ADP phosphorylation to generate ATP, also known as oxidative phosphorylation. GO exposure caused significant changes in gene expression associated with ETC. Because complexes I and III are major mitochondrial sites of ROS generation, these gene expression changes suggest ETC perturbation that may increase ROS production [74]. This pattern is consistent with the accumulation of mitochondrial ROS observed in this study. DEGs encoding complex V (ATP synthase) were significantly downregulated, suggesting potential impairment of ATP synthesis. In glycolysis/gluconeogenesis, phosphofructokinase (PFK) and pyruvate kinase (PK) are rate-limiting enzymes that control glycolytic flux. Upregulation of *PFK* and *PK* may reflect a compensatory response to maintain ATP supply under GO stress [75,76]. The pentose phosphate pathway can generate NADPH to support antioxidant defenses, and glucose-6-phosphate dehydrogenase (G6PD) is the rate-limiting enzyme. Upregulation of *G6PD* suggests the pentose phosphate pathway activation, potentially increasing NADPH availability for ROS scavenging [77]. Mitochondrial dysfunction may also disrupt other metabolic pathways. Nitrogen metabolism is ATP-demanding; ATP limitation can disrupt nitrogen metabolic balance, while nitrogen-derived metabolites can in turn influence ATP production, further perturbing energy homeostasis. Carbonic anhydrase (CA) contributes to nitrogen metabolism by regulating intracellular pH through catalysis of CO_2_ and HCO_3_^−^ interconversion [78]. Downregulation of *CA* suggests that the pH homeostasis may be perturbed under GO exposure [79]. Insect cuticular hydrocarbons (CHCs) originate from fatty acid metabolism. Reduced expression of *ACACA* and *ACSL* suggests suppressed synthesis of malonyl-CoA and long-chain acyl-CoA, which may constrain CHC biosynthesis and compromise the cuticular barrier [80]. Collectively, these results indicate that GO may impair ATP production by disrupting oxidative phosphorylation, thereby imposing energetic stress on ants. This energetic stress may secondarily destabilize metabolic networks involved in biosynthesis and oxidative defense.

## 5. Conclusions

This study provides the first evidence that GO exerts toxicological effects in social insects, using ants as a model group with key roles in terrestrial ecosystems. GO exposure induced oxidative damage, impaired mitochondrial function, altered gut microbiota composition, and changed genome-wide transcriptional profiles. Together, these multi-level perturbations across physiological, microbial, and molecular scales may reduce stress tolerance and compromise colony performance, thereby potentially weakening the ecosystem services provided by ants. These results highlight the underestimated ecotoxicological hazards of GO dispersion while offering mechanistic foundations for assessing its impacts across trophic levels.

## Figures and Tables

**Table 1:** Estimated alpha diversity for microbial communities in the control and GO groups.

Sample Group	ACE	Chao1	Simpson	Shannon
Control	1047.63 ± 180.47	999.06 ± 88.42	0.94 ± 0.057	6.96 ± 0.66
0.1%	1031.03 ± 18.89	1091.54 ± 33.48	0.98 ± 0.008	7.90 ± 0.31
0.3%	1004.57 ± 92.29	1047.59 ± 115.19	0.98 ± 0.002	8.11 ± 0.12

Values are presented as mean ± SD. Control: control group, 0.1%: 0.1% GO, 0.3%: 0.3% GO.

## Data Availability

The original contributions presented in this study are included in the article/Supplementary Materials. Further inquiries can be directed to the corresponding authors.

## Supplementary Materials

The following supporting information can be downloaded at: https://www.mdpi.com/article/10.3390/insects17010104/s1, Table S1: Primers used in this study for RT-qPCR.
