# Supplementary material for: Graphene Oxide-Induced Toxicity in Social Insects: Study on Ants Through Integrated Analysis of Physiology, Gut Microbiota, and Transcriptome
Source: Insects. 2026 Jan 16;17(1):104. doi: 10.3390/insects17010104 (PMC12841737; doi:10.3390/insects17010104)
Supplement: Supplementary file 1 [file insects-17-00104-s001.zip › Table S1.pdf]

Table S1. Primers used in this study for RT-qPCR

Gene Name	Primer Sequences (5'~3')
rpl18	F: GCTCTATCGGTTCCTGGCAAGAAG<br>R: CACAGTTCTCCCGTCCAGGTTTC
TRINITY_DN137_c0_g2_i1	F:ATTCAACTCCAACATCGTGTCGTC<br>R: CCTGCTGCACCACCAGTTATTAG
TRINITY_DN13231_c0_g1_i1	F: CCAAGTTGCCTTCCGACAAAAGTG<br>R: GCACAGGTTGCGATTACGTCATTC
TRINITY_DN813_c0_g1_i1	F: ATGAGGATTTGCCTGCAATCTCG<br>R: CGTAGCGTTGTCCTGTCTTGTG
TRINITY_DN16150_c0_g21	F: AGCGCATTCGTAGTGACATCTGG<br>R: CGAAGCTGCTTAAGTCGCGTTTAG
TRINITY_DN6000_c0_g1_i1	F: CTCGCAACTCGGTTACCCAAAGG<br>R: ATCGCCGCATCGTGTCTCTTTAAG
TRINITY_DN4292_c0_g1_i2	F: GCTTCTACTTGTATTTGCCGTGATG<br>R: GCCGTCCTTCGCTCATTGTAC
TRINITY_DN3203_c0_g1_i1	F: ACGCCGGAGATGATACGAGAAG<br>R: TGCCACCTGGGATACGATGTAAC
TRINITY_DN2683_c0_g1_i6	F: CTGGTTTTATGGGCAAGGTCCTC<br>R: TCAAGTCGATTGTCGCAACTACG
